# Anaerobic Digestion of Pig-Manure Solids at Low Temperatures: Start-Up Strategies and Effects of Mode of Operation, Adapted Inoculum, and Bedding Material

**DOI:** 10.3390/bioengineering9090435

**Published:** 2022-09-03

**Authors:** Rajinikanth Rajagopal, Vaibhavi Bele, Noori M. Cata Saady, Felipe M. W. Hickmann, Bernard Goyette

**Affiliations:** 1 Sherbrooke Research and Development Center, Agriculture and Agri-Food Canada, 2000 College Street, Sherbrooke, QC J1M 0C8, Canada; 2 Department of Building, Civil and Environmental Engineering, Concordia University, Montreal, QC H3G 1M8, Canada; 3 Civil Engineering Department, Memorial University of Newfoundland, St. John’s, NL A1B 3X5, Canada; 4 Département des Sciences Animales, Faculté des Sciences de l’Agriculture et de l’Alimentation, Université Laval, Québec, QC G1V 0A6, Canada; 5 Departamento de Zootecnia, Universidade Federal do Rio Grande do Sul, Av. Bento Gonçalves 7712, Porto Alegre 91540-000, Brazil

**Keywords:** adapted inoculum, bedding, dry anaerobic digestion, high-solids, pig manure

## Abstract

The objective of this study was to obtain start-up strategies for the operation of a dry anaerobic digestion (DAD) system treating pig-manure (PM) solids at low-temperatures, and evaluate the effects of operation mode, adapted inoculum, and bedding material on the performance. A DAD system coupled with an inoculum system (two-stage DAD) was operated at 20 ± 1 °C to digest PM solids (Total Solids, TS: 27%) with wheat straw or woodchips as bedding materials (TS substrate-mixture: 45%) using a liquid inoculum. Static DAD was also operated in parallel for comparison purposes. Overall, the percolation–recirculation mode of operation was superior to the static mode; the former had more than a 3-fold increase in specific methane yield in cycle 3. Using the adapted inoculum in cycle-2 improved methane yield by 7% and 26% for cycles 1 and 3, respectively, under the percolation–recirculation mode of operation. In addition, the digestate resulting from the digestion of woodchips + PM solids had better physical characteristics than wheat straw + PM solids. Thus, anaerobic digestion of pig-manure solids at low-temperatures with appropriate start-up strategies, inoculum, and bedding material is a promising technology for transforming PM solids into biogas and using its digestate as biofertilizer.

## 1. Introduction

The increasing demand for food has influenced the way animals are raised and the amount of waste to be disposed of. In 2020, Canada produced 2.3 million tonnes of pork and housed 13.97 million hogs [1], with production expected to increase in the following years. Confined animal-feeding operations have increased the production of large amounts of waste to be managed in relatively small spaces. Thus, greenhouse gas (GHG), especially ammonia and methane emissions from pig manure, have a huge impact on the carbon footprint of Canadian pig-production systems [2]. For the swine industry to achieve carbon neutrality with net-zero emissions in the long run, it is crucial to reduce these GHG emissions through sustainable manure-management practices. As a result, confined pig production systems that store manure provide the possibility of treating manure to reduce such emissions, produce renewable energy, and use its digestate as fertilizer. The use of treated manure rather than raw manure or inorganic fertilizer is one management strategy that seems to both maintain yields while reducing emissions [3]. However, much uncertainty persists about how different biomasses and digestates interact with some manure-management variables.

Anaerobic digestion (AD) is a promising technology that can be used to obtain biogas and better post-treatment leftover digestate from manure. Liquid manure contains < 5% solids, while semi-solid and solid manure contain 5–20% and >20% solids, respectively [4]. Dry AD (>20% solids) is efficient and requires less space [5] but may require the pre-treatment of substrates or the use of formerly adapted inoculum to reduce the lag phase in the AD process. There is a significant opportunity to reduce methane emissions from manure while producing renewable energy using anaerobic digestion [6,7]. It is also an area of tremendous opportunity, as the costs associated with manure management and treatments are high. There are new incentives for the production and use of renewable energy, with a possibility of their use as fertilizers, with the anaerobic digestion process being a highly sustainable practice that reduces GHG emissions and the carbon footprint of pig production.

Although inoculating pig-manure (PM) solids with solid inoculum to digest them in a static high-solids low-temperature anaerobic digestion process is possible, it is usually not feasible to obtain a large amount of dry inoculum to start the solid-state AD on the field, which prolongs the start-up process. Replacing the solid inoculum by liquid inoculum, and performing a percolation–recirculation operation in a two-tank (solid substrate-liquid inoculum) system could speed up the process [8]. The two-stage AD system using the percolation–recirculation (P-R) mode of operation consists of two separate tanks (solid substrate and liquid inoculum reservoir). The percolation of liquid inoculum over the solid substrate and its subsequent recirculation to a liquid tank can help to adapt the inoculum for faster AD [9], increase the volumetric loading of the digester, reduce the lag phase, and shorten the time of the treatment cycle. Acclimated liquid inoculum can be stored and used as a seed source to start future AD when required.

In cold regions such as Canada, the long winters reduce the duration available for manure land application and reduce the natural transformation rate in soil [10]. Usually, the time of the year to spread manure on land is prior to and after winter [11]. Thus, improved methods for the speedy and efficient bioconversion of manure are needed, as the majority of methane emissions from manure in Canada occurs only during a short period in the summer [12], which can potentially be mitigated with targeted intervention. In such cold-weather conditions, the type of barns, outdoor storage design, manure temperature, level of dilution, presence of cleaning products, and manure-management practices are different from those observed in warmer countries. Therefore, the specific characteristics of manure and local feedstock, including biomass, must be considered in the design of anaerobic digestion process, as this factor can negatively affect the efficiency of biogas plants.

In some regions of the world, pig manure is mixed with a bedding material such as wood chips (WC) or wheat straw (WS). The manure is treated as obtained, which usually means dealing with the bedding material included in it. AD of pig manure with rice straw [13], coffee grounds [14], or mango leaves [15] has been conducted with high methane potential. The methane yield from pig manure depends on several factors such as the separation method employed, the type of bedding material used, the temperature, etc. Some values from previous studies are given in Table 1 to outline the progress regarding the AD of pig manure.

Several studies have worked on the digestion of pig manure (or co-digestion with lignocellulosic biomass) at mesophilic temperatures [23,24,25]. Higher temperatures are preferable for anaerobic digestion; however, they may also be associated with the inhibition of anaerobic digestion due to higher levels of ammonia nitrogen [26], among other problems. A specific methane yield as high as 465 mL/gVS has been achieved from the co-digestion of mango leaves and pig manure [15]. Therefore, using second-generation biomass for improving biogas yield seems promising and relatively unexplored at low temperatures, especially for the AD of pig-manure solids.

Thus, the objectives of this study were to (i) evaluate the AD of a mixture (Total Solids, TS: 27%) of pig-manure solids with WS or WC (as bedding) at low temperatures using (i) a single-stage AD system (static-mode of operation) inoculated with a liquid inoculum (TS: 6%) and (ii) a two-stage AD system (P-R mode of operation) with liquid inoculum (TS: 2%, primarily to avoid clogging); (iii) assess the two bedding materials as co-substrates and observe the post-treatment solid matrix quality; (iv) evaluate the effect of adapted inoculum on reducing the lag phase, accelerating digestion efficiency and methane production; and (v) compare the two modes of operation. Therefore, this study can provide multi-variate useful scientific information for optimizing AD operating strategies.

## 2. Materials and Methods

### 2.1. Inoculum and Substrates

The inoculum was obtained from a semi-industrial scale anaerobic digester (11.4 m^3^) treating cow feces and operating at psychrophilic (20 ± 1 °C) conditions. It was non-adapted inoculum, i.e., inoculum not adapted to pig manure. For the single-stage AD with static operating mode, the inoculum used was not diluted, thus retaining 6% TS. However, the inoculum was diluted to 2% TS for the P-R operation mode in the two-stage AD system, mainly to avoid clogging of the solid digester. Additionally, for cycle 2 of P-R mode, the same inoculum (2% TS) was used, which was then specifically adapted to pig-manure digestion. This cycle 2 of P-R mode operated in a similar fashion to other cycles, the only difference being the use of an adapted inoculum. More details of the individual cycles such as the duration of operation, co-substrates used with pig-manure solids are given in Section 2.3. The substrates fed were pig-manure solids with wheat straw or woodchip bedding as these co-substrates are commonly used by farmers in Canada with a potential use in AD, providing structural advantages to improve the percolation process and the waste-microbes contact. Pig-manure solids were obtained by collecting pig manure under animals’ tails before it was mixed with the urine and bedding material. Fresh pig-manure solids were transferred into a plastic drum, stored at 4 °C, before being fed to digesters. The wheat straw was chopped into particle sizes of 75 mm, whereas the woodchips were smaller in size (<75 mm). The physico–chemical characteristics of all substrates are presented in Table 2.

### 2.2. Physico-Chemical and Biogas Measurements

As this study was focused on determining start-up strategies, parameters such as solids content, biogas quantity, and quality were primarily used for comparative analyses. Total (TS) and volatile (VS) solids were measured for all samples. Solids on a dry weight basis, volatile fatty acids (VFA), chemical oxygen demand (COD), total ammonia nitrogen (TAN), and total Kjeldahl nitrogen (TKN) were determined following the guidelines given by the APHA standard methods [27]. Biogas production was measured daily with a calibrated electronic gas-flow-rate meter that recorded the volume of gas passing through the meter every minute at normal temperature and pressure conditions (20 °C, 1 atm). Biogas composition (CH_4_, CO_2_, H_2_S, and O_2_) was determined weekly using a gas chromatograph (Micro GC 490, Agilent Technologies, Santa Clara, CA, USA) equipped with a thermal conductivity detector (TCD) and Helium gas as the carrier gas at a flow rate of 20 mL/min. The injector and oven temperatures were 110 °C and 180 °C, respectively. Cumulative CH_4_ yield was calculated at the end of the digestion cycle, while specific methane yield (SMY) was calculated by the amount of methane produced per unit mass of volatile solids (VS) contained in the feedstock after a given amount of time under a given temperature [28].

### 2.3. Experimental Set-Up

**Figure 1:**
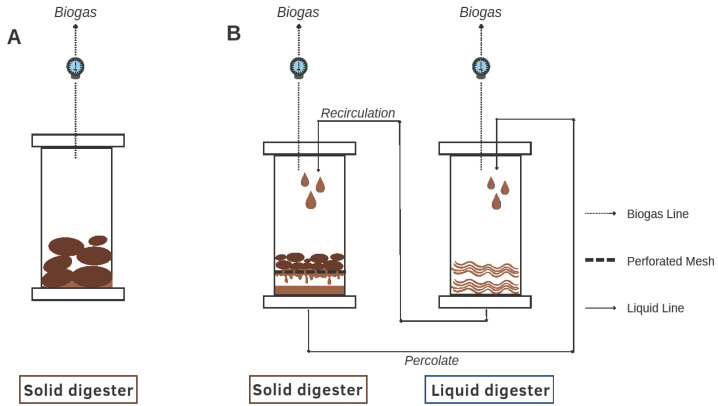
Schematic diagram of experimental set-up for (**A**) single-stage AD system with a static mode of operation and (**B**) two-stage AD system with percolate–recirculation mode of operation.

We set up 50-L cylindrical air-tight barrels and operated them as batch digesters in a temperature-controlled room (20 ± 1 °C). Digesters were fitted with two gas lines; one for purging with nitrogen gas immediately after feeding the substrate to maintain the anaerobic condition and the second to release and measure the biogas produced into the digester. A schematic diagram illustrating the details of the single-stage AD (with static mode of operation) and the two-stage AD (with P-R system) is shown in Figure 1. It is to be noted that, for static dry anaerobic digestion (DAD) system, only a single-stage digester was used, without P-R. 

The two-stage system mentioned here denotes a two-tank system interconnected with P-R wherein the methanogenesis occurs in both digesters. This is not to be confused with a two-phase system (phase separation), wherein hydrolysis and acidogenesis occur in one tank and methanogenesis occurs in the subsequent tank [29]; even though some studies use the ‘two-stage’ lexicon to indicate two phases [30,31].

**Figure 2:**
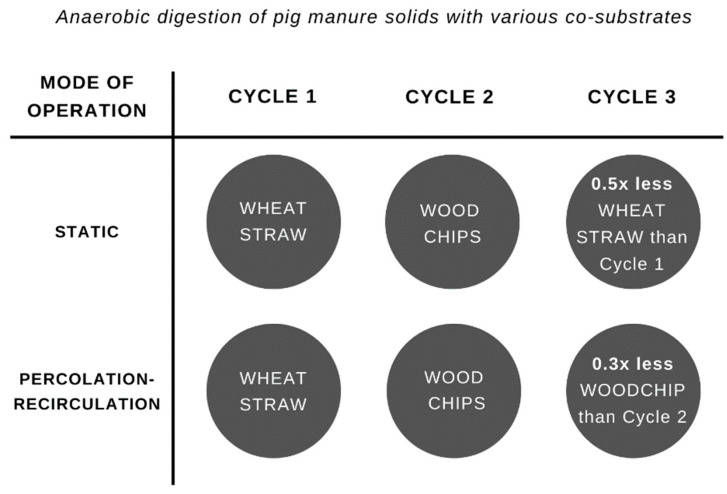
Visual representation of all experiments conducted.

To facilitate the understanding of the similarities and differences among the experiments, a visual representation is presented in Figure 2, with changes in the amount of co-substrate and bedding material represented by each cycle and the respective mode of operation. Substrates and inoculum were mixed manually for 5 min before feeding each digester.

The operating conditions for all experiments are presented in Table 3. It can be observed that the OLR and substrate-to-inoculum (S:I) ratio gradually increased as the study progressed. 

## 3. Results and Discussion

Cumulative biogas production, biogas production rate, and specific biogas production per kg of feedstock by cycle and percolation mode are given in Table 4. Since pig-manure solids are the main contributor to specific methane yield (SMY), their values are presented as a function of the amount of volatile solids present in the pig manure, i.e., the cumulative methane volume produced in liters per gram of VS_PM fed_; or based on the VS present in the feedstock (pig-manure solids and co-substrate), i.e., the cumulative methane volume produced in liters per gram of VS_fed_. The results are presented in the following sequence: (3.1) performance under a single-stage static mode of operation during cycles 1, 2, and 3; (3.2) performance under two-stage percolation-recirculation mode of operation during cycles 1, 2, and 3; (3.3) effect of adapted inoculum; (3.4) effect of mode of operation; and (3.5) bedding-material biodegradability and post-treatment digestate quality.

### 3.1. Specific Methane Yield from the Static Mode of Operation

In this study, liquid inoculum (TS: 6%) was used; however, no P-R was performed. It was then called the static mode of operation. The digestion performance was investigated in terms of SMY results from all experiments. By observing the static mode values from Table 4, it can be seen that cycle 1 gave the highest yield of 0.58 L/g VS_PM fed_ while, during cycle 2, the yield was the lowest, only 0.057 L/g VS_PM fed_.

OLR and S:I ratio were gradually increased and lowered, respectively, as the cycles under this mode of operation progressed. At the end of cycle 1, the digestion of pig-manure solids and WS yielded a SMY of 0.295 L/g VS_fed_. This result aligns with previous findings that conducted the co-digestion of pig manure with rice straw at mesophilic temperatures, wherein a SMY of 0.236 L/gVS was obtained without any pre-treatment, with a lag phase of 2.43 days [13]; both results are expressed in terms of the vs. present in the feedstock.

The quality of the biogas, as defined by its methane content, increased from none at day 0 to 55% on day 10 and remained stable at this level until the end of the cycle on day 49. It was also noted that wheat straw required a longer time to biodegrade. Its contribution to the methane yield was low initially; a hypothesis would be that the initial methane production came mainly from pig-manure solids. Lignocellulosic biomass such as wood or straw, in general, requires long time to biodegrade due to the presence of cellulose and lignin in its cell wall rendering it recalcitrant. Wheat straw contains a much higher C (in terms of total COD) and higher solids than animal manure, along with cellulose and hemicellulose accounting for ~90% of the fiber components, whereas this value is ~77% in cow feces [28]. In an interesting study, a lower amount methane was yielded from mesophilic AD of mango leaves (0.157 L/gVS) than that from pig manure (0.281 L/gVS) [15] for the same reason. Moreover, Zhong et al. [13] also found a 62.2%, 59.6%, and 33.8% reduction in the lignin, cellulose, and hemicellulose content, respectively, after the substrate (pig manure with rice straw) underwent biological pre-treatment.

During cycle 2, AD proceeded with a lag phase of approximately 10 days. The contribution of wood chips to the methane yield was initially low for the aforementioned reason. The maximum SMY was obtained on day 40 was 0.057 L/g VS_PM fed_. The reasons for the lower SMY compared to cycle 1 could include the variations in the duration of cycle and co-substrate material and quantity. The quality of the methane increased from 0 on day 0 to 30–40% towards the end of the study. A lower methane value (than the 2-stage P-R system operated during this same cycle, discussed more later) depicts that either the methanogenesis or hydrolysis or both were delayed. Based on the results of cycles 1 and 2, an attempt was made to reduce the amount of WS to about 50%, mainly to validate the performance of the AD with less bedding and, thus, to use more pig-manure solids to have a higher biogas-production rate.

Cycle 3 consisted of pig-manure solids and wheat straw and operated for just 30 days. This experiment was compared to cycle 1 regarding the co-substrate quantity. A total of 50% less wheat straw was used (0.61 kg in cycle 1 and 0.31 kg in cycle 3). Initially, it was fed with 3.5 L of inoculum (adapted to cow manure, 6% TS), as the performance was low in terms of methane production and quality (only 22% CH_4_ during days 7–14). Then, 2.5 L of additional inoculum was added to see if it would improve the performance of the static digester. The methane quality improved 52%, with SMY 0.09 L/gVS_fed_ or 0.133 L/g VS_PM fed_ obtained after 30 days of operation. This value is still comparable to the cumulative biogas production of ≈120 Nm^3^/t VS (or 0.120 NL/gVS) produced under a static mode of operation, i.e., no P-R, although it must be noted that it was AD of pig slurry and straw (dry matter of starting mixture: 20.7 ± 1.3%) under mesophilic conditions [16]. The feasibility of high-solid, dry AD at low temperatures was demonstrated in a previous study on high-rate dry AD of cow feces and wheat straw (feed TS: 35%) with the highest SMY of 151.8 ± 7.9 N L CH_4_/kg VS fed [28].

**Figure 3:**
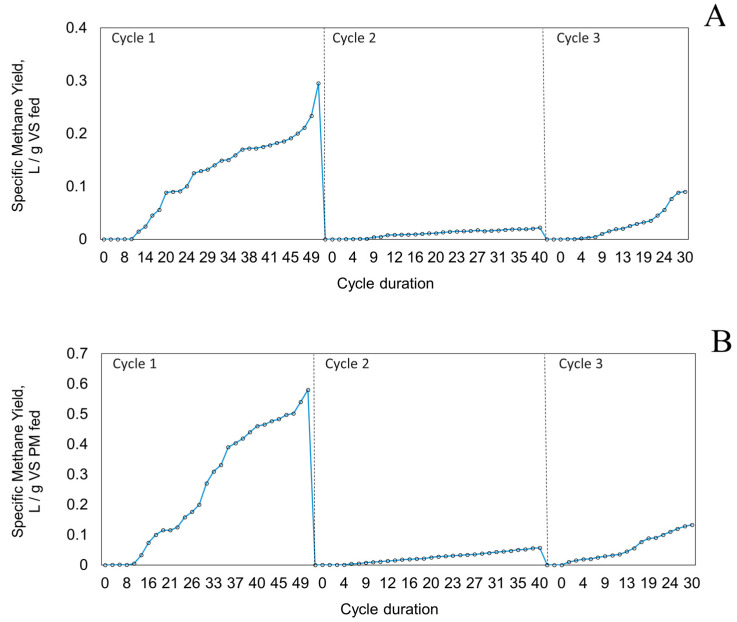
Specific methane yield profile for the static mode of operation in based on (**A**) volatile solids in feedstock mixture and (**B**) volatile solids in pig-manure solids.

Additionally, if cycles 2 and 3 from this static mode are compared (Figure 3); while both yields were smaller, cycle 3 performed slightly better than cycle 2 in terms of SMY; it is not certain whether the adapted inoculum or the amount of co-substrate (used with pig-manure solids) had more effect on it.

### 3.2. Specific Methane Yield from the Two-Stage Percolation–Recirculation Mode of Operation

For this system, a major part of the VFAs from the solid digester were washed out as percolate and recirculated to the liquid inoculum digester after P-R was completed [8], with biogas being generated from the liquid digester as well [7]. The methane yield reported here includes both digesters. Under the P-R mode of operation, the highest yield of 0.535 L/g VS_PM fed_ was obtained during cycle 2, while the lowest was 0.423 L/g VS_PM fed_ from cycle 3, as presented in Table 4 which also lists brief remarks for each cycle.

During cycle 1, AD with P-R proceeded with a lag phase of approximately 6 days. The maximum SMY obtained at day 49 was 0.249 L/gVS_fed_ and 0.500 L/g VS_PM fed_ with initial methane production from pig-manure solids; the wheat straw contribution began later. The methane content in the biogas increased from 0 on day 0 to 50% on day 21 and remained stable at this level until the end of the cycle on day 49.

For digesters operating under P-R mode and digesting a mixture of pig-manure solids and wood chips during cycle 2, there was no lag phase, unlike the one observed during cycle 1. The maximum SMY values obtained on day 40 were 0.213 L/g VS_fed_ and 0.535 L/g VS_PM fed_—the highest among all the experiments of P-R mode in this study, perhaps due to the effect of adapted inoculum. Based on these values, cycle 3 was fed with 30% less WC.

**Figure 4:**
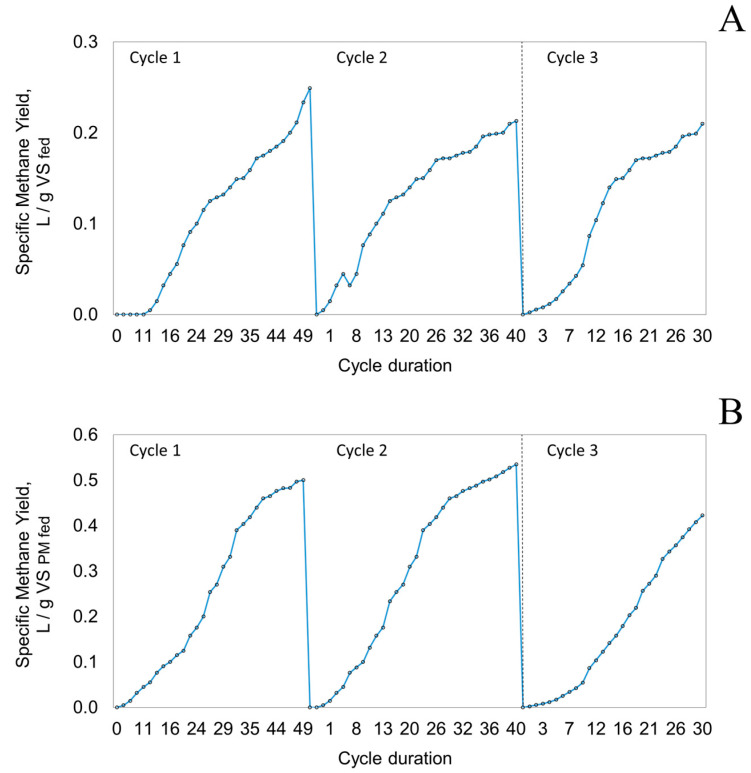
Specific methane yield profile for the percolation–recirculation mode of operation based on (**A**) volatile solids in feedstock and (**B**) volatile solids in pig-manure solids.

For cycle 3, the pig-manure solids and woodchips mixture was digested for 30 days. The co-substrate quantity was compared to that of cycle 2, with 0.66 kg of woodchip bedding being used (30% less than the 0.95 kg used in cycle 2 P-R mode of operation). The methane content was in the range of 62% and SMY 0.210 L/g VS_fed_ and 0.423 L/g VS_PM fed_, which is still close to the values of cycle 1 (49 days) and cycle 2 (40 days), which operated longer (Figure 4).

In our previous studies conducted using the P-R strategy, the highest SMY obtained was 0.14–0.16 L/g of VS_fed_ for the dry AD of poultry litter and hay bedding (feed TS: 68.6%) at low temperatures, along with an adapted liquid inoculum for P-R [7]; the study was conducted to obtain basic design criteria for the start-up of dry AD (feedstock had a CODt/TKN ratio of 26.2 and free ammonia nitrogen, FAN was 7 g/kg).

### 3.3. Effect of Adapted Inoculum

An adapted inoculum can enrich methane production, especially in the P-R mode of operation, by improving the substrate–microbe interactions [7]. A higher methane yield was obtained when the ammonia-acclimatized inoculum was used in a study to determine the methane potential of co-digesting cattle manure and microalgae [9]. The bio-augmentation of ammonia-tolerant methanogenic culture resulted in a higher methane production when used in an ammonia-induced inhibited AD process in a CSTR [32]. For an easier understanding of the effect of adapted inoculum in this study, the variables considered in the experiments are shown in Table 5. It can be noticed that the inoculum particularly adapted to pig manure was only used in cycle 2 of the P-R mode of operation.

The effect of the adapted inoculum showed some incremental value in the P-R mode of operation, as observed from the SMY values (L/g VSPM fed) in cycle 2. The woodchips used in cycle 2, despite its higher OLR and S:I ratio and lower operational period than cycle 1 under the P-R mode, had a greater methane yield; hence, the use of the adapted inoculum produced a positive output. For the same P-R mode, in cycle 3, smaller amounts of WC were fed with a shorter operational period compared to cycle 2. On the other hand, the OLR and S:I ratio were higher in cycle 3. However, for a given operating period of 30 days, cycle 3 yielded similar results to cycle 2, even with less bedding and liquid inoculum.

### 3.4. Effect of Mode of Operation

Overall, greater methane yields from the P-R mode of operation were observed compared to the static mode of operation. For the two-stage system, a major part of VFAs from the solid digester (containing pig-manure solids and bedding material) were washed to the liquid inoculum digester through the leachate (percolate), whenever percolation–recirculation was performed [8]. Hence the methane production reported in this study is the combination of both solid and liquid digesters. The VFA transfer enhanced the richness of the microbes in the liquid digester at the end of every cycle, such that it is expected to improve the solid digestion performance over time. In cycle 1, the static mode performed slightly better than the P-R mode as observed by their methane yields. A more consistent pattern is observed in cycles 2 and 3, where the P-R mode out-performed the static mode significantly.

An interesting study performed solid-state AD of pig slurry and straw with different P-R rates (1x, 2x, 4x per day) and without P-R (i.e., static) [16]. At all P-R rates, the performance was better than the static system. Additionally, at a frequency of 4x percolation per day, the highest SMY and organic-N content were observed with a lower C/N ratio among P-R rates. In fact, this is an even lesser C/N ratio than the static mode of operation. A low C/N ratio was advocated from an agronomic point of view (as for agricultural use digestate C/N ratio < 25) and the lowest TAN are desirable.

### 3.5. Bedding Material and Post-Treatment Digestate Quality

In the present study, woodchips as bedding material performed better than wheat straw when comparing co-substrates. Considering the SMY profiles from cycles 1 and 2 under the P-R mode of operation, WC seemed more biodegradable than WS. The smaller size and slightly larger amount of woodchips (0.95 kg; 0.61 kg WS was used in cycle 1) also allowed a better percolation rate, promoting greater waste–microbe interaction and biodegradation.

The characteristics of the final digestate residue at the end of cycle 1 are given in Table 6. In all cases, the digestate was an odorless solid matrix that was dried out (to a total solids content of 75%) by leaving it for 2 days in the ambient atmosphere.

Static digestate effluent C/N ratio represented as TCOD/FAN was 8.2 (this same ratio was 21.4 for raw pig-manure solids); while C/N expressed as TCOD/TKN of the effluent was 1.4 (for raw pig-manure solids, it was 5.6). The decrease in C/N ratio in the digestate indicates (i) an organic C loss due to its anaerobic degradation and subsequent conversion to biogas, and (ii) an increase in organic N content which could be beneficial from an agricultural standpoint as the increase in TKN with a concurrent decrease in FAN means that more organic N is recovered, which could be used as a biofertilizer.

## 4. Conclusions

The anaerobic digestion of pig-manure solids at low temperatures with appropriate start-up strategies, mode of operation, inoculum, and bedding material is a promising technology for transforming pig manure into biogas or using it as biofertilizer; it is an alternative to be used by the swine industry on the path towards carbon neutrality. In this study, the effect of the mode of operation and an adapted inoculum were observed. High methane yields were obtained from all experiments under the percolate–recirculation mode. The use of adapted inoculum in P-R mode allowed the reduction of the operation time, increasing the organic loading rate and substrate-to-inoculum ratio. This effect of adapted inoculum was not observed under the static-mode experiments; nevertheless, it gave the highest methane yield among all experiments, indicating the possibility of feasible static AD. The findings from this study provide important validation to conduct further experiments with an in-depth analysis of all physico–chemical parameters. For future studies, statistical analyses to observe correlations between different variables and assess the significance of results will propagate an even better understanding of AD systems.

## Figures and Tables

**Table 1:** Summary of studies on biogas from solids separated from manure.

Manure Type	Separation Method	TS (%)	Temperature (°C)	SMY	Remarks	Reference
Pig manure (co-digestion with mango leaves)	Sieved through 2 mm pore size	28.29 ± 0.08	37 ± 1	465 mL/gVS (at mango leaves:pig manure mix ratio 1:3) 281 mL/gVS (only pig manure)	- Substrate: inoculum (%) = 20:100 - Digestion period–33 days - Mixing speed 100 rpm	[15]
Pig slurry and straw	None	20.7 ± 1.3	35 ± 2	120 mL/gVs (static mode–no recirculation) 240.8 mL/gVS (P-R mode)	- Two modes of operation studied: static and P-R with different P-R rates. - Overall P-R mode performed better. - Post-AD digestate residue was high in organic-N.	[16]
Liquid pig manure (co-digestion with spent coffee grounds)	None	26.5 ± 5.3 g/L	37	323 ± 29 mL/gVS (co-digestion)	- Manure: coffee grounds mix ratio not specified - Operational period 70 days - Substrate: inoculum 1:1 - Co-digestion produced synergistic effect	[14]
Pig-manure solids (co-digestion with dried grass)	Decanter centrifuge, alum, and polyacrylamide flocculant (PAM) diluted with water	28.2	35 ± 1	252.5 mL CH_4_/g VS	- Retention time = 60 days - OLR = 1 kg VS/m^3^/d - Manure solids: dried grass % ratio = 80:20	[17]
Pig-manure solids (acidified)	Drum screen separation 1 mm screen size and 7 rpm speed	10.60	35	319.3 ± 1.9 L/kg VS	Retention time = 90 days	[18]
Sow manure solids (acidified)	Screw press separation with mm screen size and mm plate tension					
Pig manure (co-digestion with rice straw)	None	N.A.	35 ± 0.5	≈342 mL/gVS (pre-treated) ≈236 mL/gVS (not pretreated)	- Operation period 15–20 days and 30 rpm (mixing) - Substrates were pretreated with cellulolytic microflora	[13]
Pig manure	Mechanical sieve 0.25 mm pore size	11.4	35	251 L CH_4_/kg COD_fed_		[19]
Pig manure	Flocculation with coagulant aid	13.6	35	110 L CH_4_/kg VS		[20]
				155 L CH_4_/kg COD_fed_		
				135 L CH_4_/kg vs. _fed_		
		9.9		157 L CH_4_/kg COD _fed_		
Pig manure	Centrifugation	32		161 to 186 L CH_4_/kg VS	Retention time = 60 days	[21]
Pig manure	Coagulation and flocculation	12.2		392 to 404 L CH_4_/kg VS	Retention time = 60 days	
Pig manure	Centrifugation	29	35	261 L CH_4_/kg VS	Biodegradability = 51%	[22]
Pig manure	Centrifugation	31	35	159 L CH_4_/kg VS	Biodegradability = 30%	
Pig manure	Chemical treatment	28.5	35	247 L CH_4_/kg VS	Biodegradability = 48%	

**Table 2:** Physico-chemical characteristics of pig-manure solids and wheat straw.

Parameters	Pig-Manure Solids	Wheat Straw	Woodchips
Dry matter (DM, %)	26.9	90	90
Volatile solids (VS, %)	23	85	85
Fixed solids (FS, %)	--	15	15
Total volatile fatty acids (TVFA, g/L)	23.1	NA	NA
pH	6.6	NA	NA
Total chemical oxygen demand (TCOD, g/L)	216.3	1079	NA
Total Kjedahl nitrogen (TKN, g/L)	38.5	NA	NA
Ammonia (NH_3_, g/L)	10.1	NA	NA

**Table 3:** Operating conditions and variables for all experiments in the three operation cycles.

	**Cycle 1**		**Cycle 2**		**Cycle 3**	
Substrates used	PM solids + WS	PM solids + WS	PM solids + WC	PM solids + WC	PM solids + WS	PM solids + WC
Mode of operation	Static DAD	2-stage P-R	Static DAD	2-stage P-R	Static DAD	2-stage P-R
Inoculum used	Yes *	Yes *	Yes *	Yes **	Yes *	Yes *
PM solids + co-substrate (kg)	2.32 + 0.61	2.32 + 0.61	2.32 + 0.95	2.32 + 0.95	2.38 + 0.31	2.38 + 0.66
Liquid inoculum (kg)	12	36	12	36	6	6
OLR (g VS/kg inoculum/day)	3.1	1.0	4.12	1.4	4.5	4.3
S:I ratio	0.24	0.08	0.27	0.09	0.45	0.51
Feedstock TS (%)	40.0	40.0	45.9	45.9	34.3	33.3
Inoculum TS (%)	6	2	6	2	6	6
Operation period (days)	49	49	40	40	30	30

* obtained from the digester treating cow manure ** obtained from the digester treating cow manure but specifically adapted to pig manure at a later stage.

**Table 4:** Specific methane yield values for each cycle and both modes of operation.

	**Single-Stage AD with Static Mode**			**Two-Stage AD with P-R Mode**		
Cycle number (duration in days)	1 (49)	2 (40)	3 (30)	1 (49)	2 (40)	3 (30)
Total biogas production (L)	NA	100	170	NA	550	410
Biogas production rate (L/d) *	NA	2.5	5.7	NA	13.8	13.7
Biogas production (L/kg raw feedstock) **	NA	30.58	63.19	NA	168.19	134.86
SMY (L/g VS_fed_)	0.295	0.022	0.090	0.249	0.213	0.210
SMY (L/g VS_PM fed_)	0.580	0.057	0.133	0.500	0.535	0.423
Remarks	WS required longer time, and its contribution to CH_4_ production began later.	Non-adapted inoculum use explains low SMY.	50% less WS and inoculum volume were used compared to cycle 1.	WS required longer time to contribute to CH_4_ production.	Adapted inoculum improved the SMY value.	Inoculum recirculation stopped after 20 days which explains the slight drop in SMY. 30% less WC used compared to cycle 2.

* measured at normal temperature and pressure conditions (20 °C, 1 atm). ** per kg raw feedstock amount contains pig-manure solids and either wheat straw or wood chips.

**Table 5:** Parameters considered to observe the effect of the adapted inoculum.

Mode		Static			P-R		
Cycle		1	2	3	1	2	3
Co-substrate	WS	X		X	X		
	WC		X			X	X
Inoculum	Adapted					X	
	Non-adapted	X	X	X	X		X
SMY	L/g VS_PM fed_ up to 30 days	0.28	0.05	0.13	0.40	0.48	0.42

P-R = percolation-recirculation mode of operation; WS = wheat straw; WC = woodchips; SMY = specific methane yiled; X indicates used in the experiment.

**Table 6:** Digestate physico–chemical characteristics at the end of cycle 1.

Physico–Chemical Parameters	Digestate (from Static Mode of Operation)	Digestate (from P-R Mode of Operation)
Dry matter (DM, %)	12.4	12
Volatile solids (VS, %)	10	9.87
Fixed solids (FS, %)	2.4	-
Total volatile fatty acids (TVFA, g/L)	744	841
pH	8.2	8.1
Total chemical oxygen demand (TCOD, g/L)	66.96	117.72
Ammonia (NH_3_-N, g/L)	8.18	-
Ammonium (NH_4_, g/L)	12.3	4.99
TKN (g/L)	48.1	34.4

## Data Availability

Data may be available from authors upon reasonable request.

## Abbreviations

AD	Anaerobic Digestion
PM	Pig Manure (solids)
WS	Wheat Straw
WC	Wood Chips
DAD	Dry Anaerobic Digestion (static)
P-R	Percolation–Recirculation or Percolate–Recirculation
SMY	Specific Methane Yield
FAN	Free Ammonia Nitrogen
TAN	Total Ammonia Nitrogen
VFA	Volatile Fatty Acids
TCOD	Total Chemical Oxygen Demand (COD)
TKN	Total Kjeldahl Nitrogen
C/N ratio S:I ratio	Carbon-to-Nitrogen ratio Substrate-to-Inoculum ratio
